# Resolving inconsistent effects of tDCS on learning using a homeostatic structural plasticity model

**DOI:** 10.3389/fnetp.2025.1565802

**Published:** 2025-07-07

**Authors:** Han Lu, Lukas Frase, Claus Normann, Stefan Rotter

**Affiliations:** ^1^ Bernstein Center Freiburg, University of Freiburg, Freiburg, Germany; ^2^ Faculty of Biology, University of Freiburg, Freiburg, Germany; ^3^ BrainLinks-BrainTools, University of Freiburg, Freiburg, Germany; ^4^ Centre National de la Recherche Scientifique, Université de Strasbourg, Institut des Neurosciences Cellulaires et Intégratives UPR3212, Strasbourg, France; ^5^ Department of Psychosomatic Medicine and Psychotherapy, Medical Center, Faculty of Medicine, University of Freiburg, Freiburg, Germany; ^6^ Department of Psychiatry and Psychotherapy, Medical Center, University of Freiburg-Faculty of Medicine, Freiburg, Germany; ^7^ Center for Basics in Neuromodulation, Freiburg, Germany

**Keywords:** tDCS, motor learning, homeostatic structural plasticity, spiking neural network, cell assembly

## Abstract

**Introduction:**

Transcranial direct current stimulation (tDCS) is increasingly used to modulate motor learning. Current polarity and intensity, electrode montage, and application before or during learning had mixed effects. Both Hebbian and homeostatic plasticity were proposed to account for the observed effects, but the explanatory power of these models is limited. In a previous modeling study, we showed that homeostatic structural plasticity (HSP) model can explain long-lasting after-effects of tDCS and transcranial magnetic stimulation (TMS). The interference between motor learning and tDCS, which are both based on HSP in our model, is a candidate mechanism to resolve complex and seemingly contradictory experimental observations.

**Methods:**

We implemented motor learning and tDCS in a spiking neural network subject to HSP. The anatomical connectivity of the engram induced by motor learning was used to quantify the impact of tDCS on motor learning.

**Results:**

Our modeling results demonstrated that transcranial direct current stimulation applied before learning had weak modulatory effects. It led to a small reduction in connectivity if it was applied uniformly. When applied during learning, targeted anodal stimulation significantly strengthened the engram, while targeted cathodal or uniform stimulation weakened it. Applied after learning, targeted cathodal, but not anodal, tDCS boosted engram connectivity. Strong tDCS would distort the engram structure if not applied in a targeted manner.

**Discussion:**

Our model explained both Hebbian and homeostatic phenomena observed in human tDCS experiments by assuming memory strength positively correlates with engram connectivity. This includes applications with different polarity, intensity, electrode montage, and timing relative to motor learning. The HSP model provides a promising framework for unraveling the dynamic interaction between learning and transcranial DC stimulation.

## Graphical Abstract

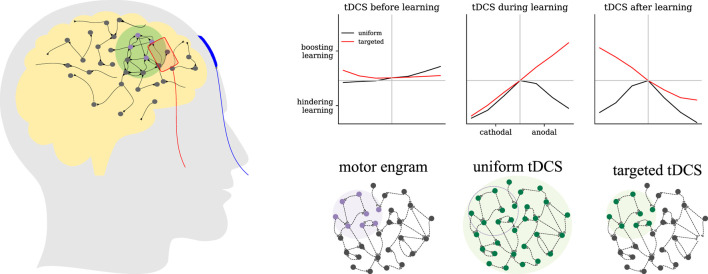

## Introduction

Transcranial direct current stimulation (tDCS) is a promising non-invasive brain stimulation with a long history (Sarmiento et al., 2016; Majdi et al., 2022; Burkhardt et al., 2023). By applying a weak direct current (1– 2 mA ) to the brain through external surface electrodes attached to the scalp, although not demonstrated in all studies, tDCS can instantly modulate the firing activity of active neurons by influencing the release of vesicles from presynaptic sites (Rahman et al., 2013; Chakraborty et al., 2018; Vasu and Kaphzan, 2021; Vasu and Kaphzan, 2022) or polarizing the membrane potential (Bikson et al., 2004; Lang et al., 2005; Radman et al., 2006; Radman et al., 2007; Radman et al., 2009a; Radman et al., 2009b; Vöröslakos et al., 2018) and significantly altering network calcium activity if delivered in short pulses of direct current electric field (dcEF) stimulation (Shaner et al., 2023). However, changes in neural excitability, synaptic plasticity, and behavioral effects persist up to hours or even days after stimulation has been terminated (Nitsche and Paulus, 2001; Laste et al., 2012; Palm et al., 2016; Kronberg et al., 2017; Latchoumane et al., 2018; Yang et al., 2019). Exploiting these direct and indirect effects, tDCS is increasingly used in experimental and clinical settings to modulate brain functions (Fregni and Pascual-Leone, 2007; Flöel, 2014; Kuo et al., 2014; Wang et al., 2021; Balzan et al., 2022; Pergher et al., 2022), modulate gene expressions (Kim et al., 2017), and treat diseases (Nitsche et al., 2003a; Baeken et al., 2016; Buch et al., 2017; Kim et al., 2017; Thibaut et al., 2017; Morya et al., 2019; Ghasemian-Shirvan et al., 2020; Jog et al., 2023). With increased usage and growing diversity of tDCS studies, it is now often applied in patients and healthy volunteers that are concurrently engaged in other tasks, such as learning (Reis et al., 2008; Clark et al., 2012; Coffman et al., 2014; Orban de Xivry and Shadmehr, 2014; Buch et al., 2017; Greeley et al., 2022; Pergher et al., 2022; Rivera-Urbina et al., 2022).

Motor learning is the paradigm most frequently studied in tDCS, as there are established protocols and effective tests for cortical excitability (Reis and Fritsch, 2011; Saucedo Marquez et al., 2013; Ammann et al., 2016; Buch et al., 2017), as well as its great potential in rehabilitating motor skills with low adverse events in patients with Parkinson’s disease or after stroke (Alsharidah et al., 2018; O’Brien et al., 2018; Ciechanski et al., 2020; Halakoo et al., 2020; Berrigan et al., 2021; Manto et al., 2021; Duan and Zhang, 2024), *etc.* Among these studies, the cerebellum, the primary motor cortex (M1), the premotor cortex (M2), the prefrontal cortex (PFC), and the posterior parietal cortex (PPC) are the most targeted areas, where parameters such as current polarity, current intensity, electrode montage, and stimulation timing are acknowledged to have a discernible influence on the outcome of stimulation (see a recent review in Qi et al. (2022)). For example, when applying tDCS to M1 concurrently with a motor learning task, it was reported that anodal tDCS improved learning (Soekadar et al., 2015; Debarnot et al., 2019), while cathodal tDCS did not produce comparable effects (Nitsche et al., 2003b; Ammann et al., 2016) or even reduced retention (Kim et al., 2024). However, some follow-up studies that used slightly different configurations or tasks failed to replicate these effects (Saucedo Marquez et al., 2013; Ambrus et al., 2016; Buch et al., 2017; Sevilla-Sanchez et al., 2022). In addition, the strength of the after-effects on cortical excitability did not correlate well with the current intensity (Batsikadze et al., 2013; Cuypers et al., 2013; Jamil et al., 2017). The focality of stimulation depends on the electrode montage, and this is known to also influence the effect of tDCS on motor learning. Traditionally, either unilateral (with the anode on the non-dominant M1 and the cathode over the contralateral supraorbital area) or bilateral (anode over the dominant and cathode over the non-dominant M1) electrode montages are used. Interestingly, these two configurations produce different results (Goodwill et al., 2013; Naros et al., 2016). To improve focality in a specific brain area, high definition tDCS (HD-tDCS) was proposed, *e.g.*, employing a central anode surrounded by four cathodes. HD-tDCS induces a more focal electric field that was shown in recent pilot studies to facilitate motor learning (Cole et al., 2018; Iannone et al., 2022) and differentially recruits different pathways to M1 (Iannone et al., 2022). However, as more and more studies emerge, it becomes clear that the relation between motor learning and tDCS strongly depends on the learning phase. The same tDCS protocol applied before learning, during training blocks, or after learning leads to very different results (Tecchio et al., 2010; Stagg et al., 2011; Apolin et al., 2016; Parma et al., 2021; Rivera-Urbina et al., 2022). These effects appear to differ in brain area (Rivera-Urbina et al., 2022), subject age (Greeley et al., 2022), and health condition (Simpson and Mak, 2022). Assuming that tDCS is, in principle, a perturbation of the underlying neural activity, the outcome of tDCS should be brain state dependent. However, the neural correlate of this dependency has not yet been systematically elucidated.

Synaptic plasticity was recognized as the key mechanism underlying the modulatory effects of tDCS on motor learning. Animal studies focused primarily on M1 and suggested that M1 displays strong activity-dependent plasticity (Sanes and Donoghue, 2000), and dendrite-specific spine formation was observed in motor skills training (Xu et al., 2009; Yang et al., 2014). After 3 days of *in vivo* tDCS stimulation ( 20 min per day) in M1, mice presented improved synaptic transmission and increased spine density in pyramidal neurons in layer II/III of M1, as well as improved motor performance (Barbati et al., 2020). Indirect evidence from human experiments applying tDCS in motor learning is abundant, where both Hebbian and homeostatic phenomena have been reported (Kuo et al., 2008): For example, pre-learning anodal tDCS was considered to trigger homeostatic inhibition and hinder learning performance. In contrast, tDCS concurrent with learning leads to boosting effects similar to long-term potentiation (LTP). This was interpreted as a Hebbian phenomenon. However, the explanatory power of currently used models of homeostatic and Hebbian plasticity is limited (Antal et al., 2008). In addition, those models do not account for spine turnover. We recently showed that the homeostatic structural plasticity (HSP) model can provide a theoretical framework to explain the long-lasting after-effects of tDCS (Lu et al., 2019), repetitive transcranial magnetic stimulation (rTMS) (Anil et al., 2023), and repetitive optogenetic stimulation (Lu et al., 2021). According to the HSP rule, neurons continuously grow synaptic elements (boutons and spines) and specifically form new synapses if their neural activity falls below the homeostatic setpoint (Van Ooyen, 2011; Gallinaro and Rotter, 2018; Lu et al., 2019; Lu et al., 2021; Gallinaro et al., 2022). The HSP rule can also implicitly have associative properties (Hebbian-like) on top of its primary function to homeostatically regulate neural activity (Gallinaro and Rotter, 2018; Gallinaro et al., 2022). In line with this idea, the HSP model is considered a good candidate to explain the dynamic interaction between tDCS and motor learning.

In our current study, we conceived motor learning as the formation of new structures corresponding to new memories (“engrams”) in a recurrent network. Transcranial stimulation was assumed to alter the equilibrium between neuronal activity and network structure, leading to additional network rewiring that interferes with learning. We analyzed the effect of different combinations of stimulation parameters, such as electrode montage, DC polarity and intensity, as well as the relation to the learning phase. In summary, our model can reconcile the observed impact of DC stimulation on motor learning and reconcile seemingly contradicting experimental results. We also made predictions that have not yet been reported by human experiments. The HSP model, linking structural changes and the homeostatic regulation of neural activity, provides a systematic framework for explaining and predicting the effects of tDCS on motor learning.

## Methods

### Neuron, synapse, and network models

Numerical simulations of neural networks with homeostatic structural plasticity were used to explore the interaction between tDCS and learning. We used the same neuron model, synapse model, and network model, as published in our previous work (Lu et al., 2019; Gallinaro et al., 2022; Lu et al., 2023). The NEST simulator (Linssen et al., 2018) with parallel MPI-based computation was used to perform the large-scale neural network simulations presented here.

We used a leaky integrate-and-fire (LIF) neuron model. The cortical area M1 was conceived as an inhibition-dominated recurrent neural network of 10000 excitatory and 2500 inhibitory neurons (Brunel, 2000). All connections involving inhibitory neurons were static with a fixed weight. Recurrent synapses among excitatory neurons (E-E connections) were grown according to the HSP rule (Gallinaro and Rotter, 2018; Lu et al., 2019; Gallinaro et al., 2022). Each neuron in the network received the same Poissonian external input. After the 750s growth period, the network had established an equilibrium between neural activity and network architecture. Excitatory neurons fired asynchronously and irregularly at 8Hz and were connected with a connection probability around 9% . Details concerning neuron and network models, as well as the HSP rule, are described in the Supplementary Material.

### Motor learning protocol

In a previous work, learning and memory was simulated as an engram (Gallinaro et al., 2022). Here we followed the idea and simulated the motor learning training process in a motor engram comprising 1000 excitatory neurons ( 10% of all excitatory neurons) consistently for all experiments. The neurons were driven by Poissonian spike input at a rate of 1.5kHz with synaptic weight J=0.1mV for 150s. The input of motor learning led to a fluctuating membrane potential with a mean value $\mu =\nu_{\mathrm{ext}}\tau_{m}J_{\text{learning}}=1.50\;mV$ (Brunel, 2000). The connectivity of the motor engram $\left(\Gamma_{\text{engram}}\right)$ was conceived as the main readout that represents the strength of memory.

### Direct current stimulation protocol

A model of how tDCS affects single neurons was published and characterized in our previous paper (Lu et al., 2019). Thus, we assumed that the electric field induced by the administration of transcranial current polarizes the soma of neurons with extended nonisotropic morphology, such as pyramidal neurons (Radman et al., 2009b; Vöröslakos et al., 2018). The duration of the stimulus was 150s for all experiments. However, we tested different configurations for the polarity and intensity of the current and of the electrode montage.

In all of our experiments, the E-E connections were grown during an initial 750s growth period in the presence of background input. The motor learning protocol and/or tDCS were applied after the network had reached its structural equilibrium.

### Electrode montage

We first devised three paradigmatic scenarios to explore the impact of the electrode montage. The traditional two-electrode montage may induce a diffuse electric field that covers a volume larger than the volume engaged in motor learning. In contrast, the HD-tDCS montage induces a focal electric field that targets only a subset of neurons. In our current study, we concentrated on three extreme scenarios: uniform, targeted, and unfocused. In the uniform scenario, DC stimulation was applied homogeneously to all excitatory neurons in the network. Targeted DC stimulation was administered exclusively to the motor engram. Unfocused stimulation was an intermediate scenario, in which DC stimulation covers only half of the engram cells and the same amount of non-engram excitatory neurons.

### Relative timing of motor learning and transcranial DC stimulation

We tested three experimental conditions for each electrode montage. We applied tDCS immediately before, during, or immediately after learning input.

### Polarity and intensity of transcranial DC stimulation

For each combination of electrode montage and relative timing, we systematically varied the amplitude of tDCS to polarize the membrane potential between −0.6mV and 0.6mV . If not stated otherwise, the transcranial DC currents used in this study were generally weaker than the motor learning input (1.5mV) .

### Measurement of firing rate and connectivity

To quantify the modulatory effect of tDCS on learning, we considered the temporal evolution of neural activity and the connectivity of the motor engram. The neural firing rate was calculated from the spike count in a recording window of 5s . The mean activity of the population was estimated by averaging the firing rates of individual neurons. We used a n×n connectivity matrix $\left(A_{ij}\right)$ to represent the recurrent excitatory connections of our network, where columns and rows correspond to pre- and postsynaptic neurons. The entry $A_{ij}$ of the matrix represents the total number of synaptic connections from neuron j to neuron i . The mean network connectivity at any given time t was calculated by $\Gamma \left(t\right)=\frac{1}{n^{2}}\sum_{ij}A_{ij}$ .

### Data and code availability

All code and dataset are available here: https://github.com/ErbB4/tDCS-and-learning.git.

## Results

### Modeling motor learning with a motor engram

**FIGURE 1:**
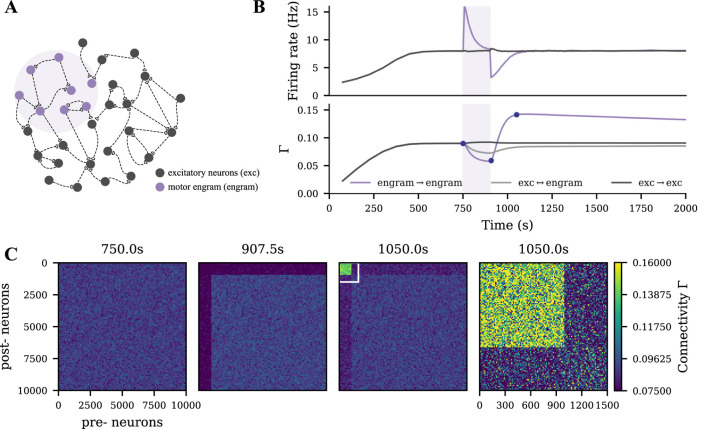
Strong motor engram formed during motor learning. **(A)** Schematic of the M1 neural network, part of which (10%) was subject to input that models motor learning. **(B)** Temporal evolution of neural activity and network connectivity (Γ) during a typical motor learning episode (purple shading). The purple and dark gray curves in the upper panel represent the firing rates of the motor engram and of the remaining excitatory neurons, respectively. The purple, dark gray, and light gray curves in the lower panel represent the intra-group connectivity of the memory engram, intra-group connectivity of the non-stimulated excitatory neurons, and inter-group connectivity between both populations. **(C)** The E-E connectivity matrix at the three time points indicated in panel B (dark blue dots), the fourth panel shows a scaling of polarities and intensities motor engram connectivity. The color scale accounts for connectivity (connection probability).

We modeled motor learning by introducing excitatory spike input to a subgroup of the excitatory neurons (Figures 1A,B, purple shading). The application and termination of motor learning input substantially altered the neural activity of stimulated neurons and induced homeostatic reorganization of synapses as previously described (Gallinaro and Rotter, 2018; Lu et al., 2019; Gallinaro et al., 2022). When the disrupted neural activity returned to the homeostatic level, the network architecture did not recover to the pre-learning state. Instead, neurons receiving motor learning input wired more with each other, but less with other non-stimulated excitatory neurons (Figure 1C). This cell assembly with elevated connectivity is referred to as a motor engram in the following.

### tDCS triggers similar but weaker network remodeling than motor learning

**FIGURE 2:**
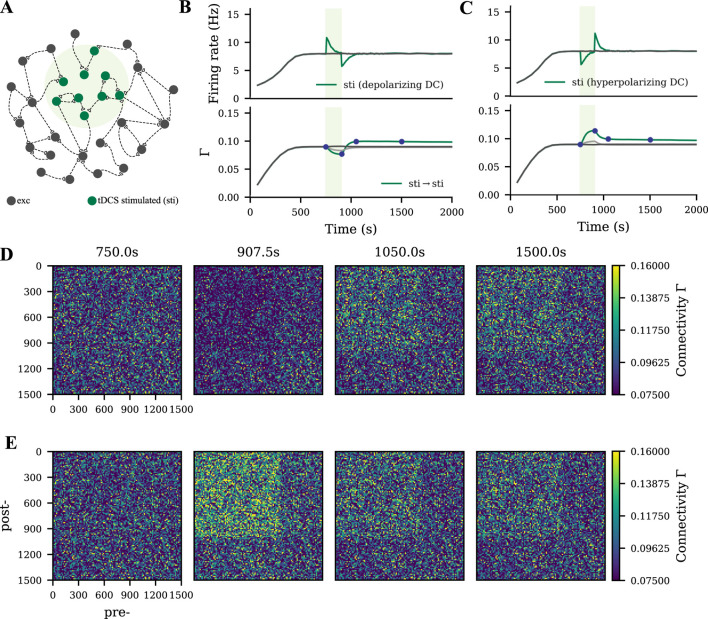
Weak cell assembly formed during tDCS. **(A)** Schematic of the M1 neural network, part of which (10%) was weakly polarized by the electrical field applied transcranially. **(B, C)** Temporal evolution of neural activity and network connectivity, when the membrane potential of neurons was depolarized or hyperpolarized during tDCS. **(D, E)** The E-E connectivity matrix at four time points indicated in panels B and C (dark blue dots). Depolarizing and hyperpolarizing tDCS triggered different processes of synaptic reorganization, but both lead to a similar persistent increase in connectivity among the stimulated neurons.

We modeled tDCS by injecting subthreshold direct current into the soma as described in our previous study (Lu et al., 2019). When the membrane potential of a subpopulation was depolarized or hyperpolarized by DC (Figures 2A–C, green shading), a similar synapse reorganization process occurred. The DC input strength that we used in the current study was relatively weaker than the learning input, so the connectivity of the tDCS-stimulated cell assembly was accordingly weaker than the motor engram (Figures 2D,E). In contrast, no resulting cell assembly was triggered when tDCS was applied globally to all excitatory neurons.

### Uniform DC stimulation weakens the motor engram

**FIGURE 3:**
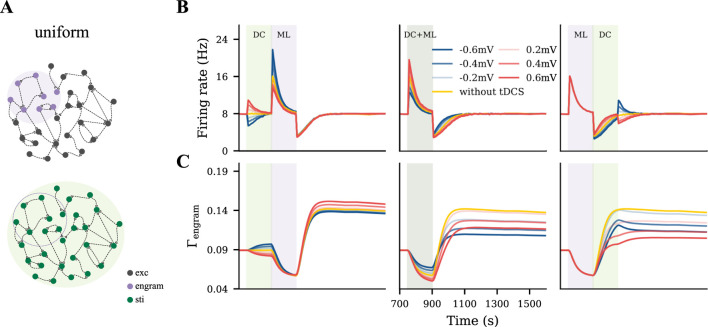
Uniform tDCS generally hinders motor learning. **(A)** Uniform tDCS was applied to the entire M1 network where the motor engram is embedded. **(B, C)** Temporal evolution of firing rate and connectivity under three different conditions: applying uniform tDCS immediately before, during, or immediately after learning input. The red and blue curves represent depolarizing and hyperpolarizing DCs, respectively. The yellow curve represents the condition without tDCS. The green and purple shaded areas represent the period of tDCS application (DC) and motor learning (ML), respectively.

To capture a rather diffusive tDCS scenario, uniform tDCS was administered to all excitatory neurons before, during, or after the learning process, with varying polarity and intensity (Figure 3A). In Figures 3B,C, we displayed the evolution of the firing rate and connectivity of the motor engram for all conditions. When applied before the learning process, uniform anodal tDCS slightly increased motor engram connectivity, while cathodal stimulation led to a reduction in connectivity. However, when applied concurrently with learning or post-learning, the uniform tDCS weakened the motor engram irrespective of the DC polarity. The weakening effects further depended on the DC intensity: Stronger tDCS resulted in lower connectivity of the motor engram. In summary, our simulation results demonstrated a divergent impact of uniform tDCS on learning, but with a strong tendency to negatively influence motor learning.

### Targeted tDCS influences motor engram in a phase- and polarity-dependent manner

**FIGURE 4:**
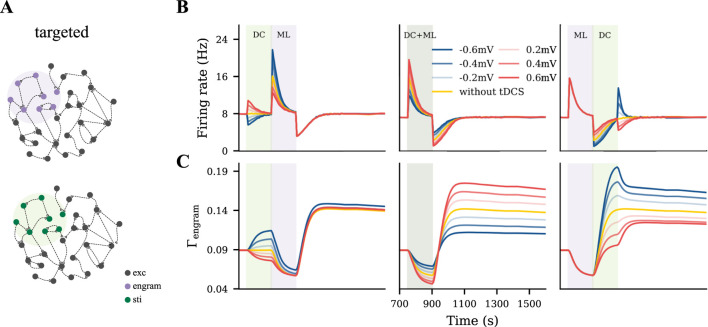
Targeted tDCS exerts opposite effects when applied at different stages of learning. **(A)** Targeted tDCS matched with the entire motor engram. **(B, C)** Temporal evolution of the motor engram’s firing rate and connectivity, when targeted tDCS was applied either immediately before, during, or immediately after the learning input.

To model a targeted focal stimulation, we applied tDCS exclusively to excitatory neurons within the motor engram before, during, and after motor learning with different polarities and intensities (Figure 4A). Our simulation results showed that the final connectivity of the motor engram strongly depended on the relative phase of motor learning and the polarity of tDCS (Figures 4B,C). When applied before motor learning, anodal and cathodal-centered tDCS slightly facilitated engram connectivity. However, when applied concurrently, anodal DC boosted the engram connectivity, whereas cathodal stimulation weakened it. Furthermore, when applied post-learning, the opposite effects were observed: cathodal DC increased the connectivity of the motor engram while the anodal DC reduced it. In summary, our simulation results suggested that the relative learning phase and polarity of tDCS should be chosen with care; targeted anodal and cathodal tDCS might exert opposite effects at different stages of motor learning.

### Unfocused tDCS and the interaction of cell assemblies

**FIGURE 5:**
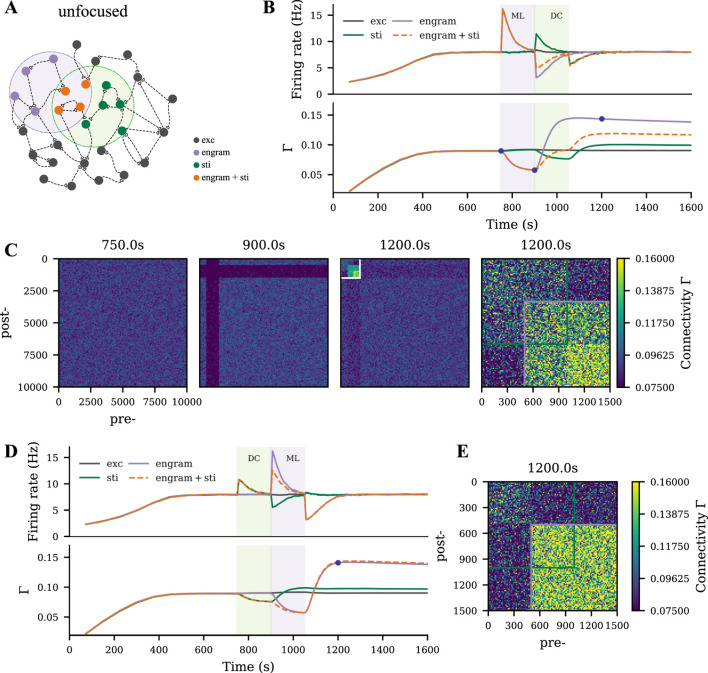
Motor engram and tDCS stimulated cell assembly interfere in case of overlap. **(A)** Schematic of the M1 neural network, where the motor learning engram and the tDCS stimulated cell assembly had 50% overlap. **(B)** Temporal evolution of firing rate and connectivity of excitatory neurons when applying tDCS after learning. The green and purple curves represent neurons that receive only tDCS or only motor learning input, respectively. The orange dashed curve reflects the overlapped half of the engram, and the dark gray curve represents the non-stimulated excitatory neurons. **(C)** Connectivity matrix at the three time points indicated in panel B. In the right panel of C, the green square indicates connectivity among the assembly of tDCS-stimulated cells, while the purple square labels the motor learning engram. **(D, E)** Temporal evolution of neural activity and network connectivity, and the resulting connectivity matrix, when tDCS was applied before motor learning.

The uniform and targeted tDCS represent two extreme conditions, in which tDCS either homogeneously covers the entire network or exclusively targets the motor engram. Neither holds in the practice of the application of tDCS in humans. So we proposed an intermediate condition, unfocused tDCS, by shifting the targeted tDCS to cover only half of the motor engram (Figure 5A). We expected the motor engram and the stimulated cell assembly to interact with each other during the dynamic stimulation process mediated by homeostatic structural plasticity. To analyze this effect, we presented two examples by applying a depolarizing tDCS at the same intensity before or after the motor learning process. In Figures 5B,D, we show the evolution of the firing rate and connectivity of four subpopulations: The overlapped half of the motor engram (orange), the non-overlapped half of the motor engram (purple), neurons receiving tDCS only (green), and the non-stimulated excitatory neurons (dark gray).

When applying the motor learning input prior to tDCS, substantial network remodeling took place to form a motor engram, while the following weak depolarizing tDCS posed the opposite dynamics to the overlapped motor engram. As a result, the connectivity of the overlapped half engram was lower than that of the non-overlapped half (Figure 5C, last panel). In contrast, when applying tDCS before learning, connectivity within the motor engram was not strongly affected, but the overlapped half ended up with higher connectivity than the non-overlapped half (Figure 5E).

**FIGURE 6:**
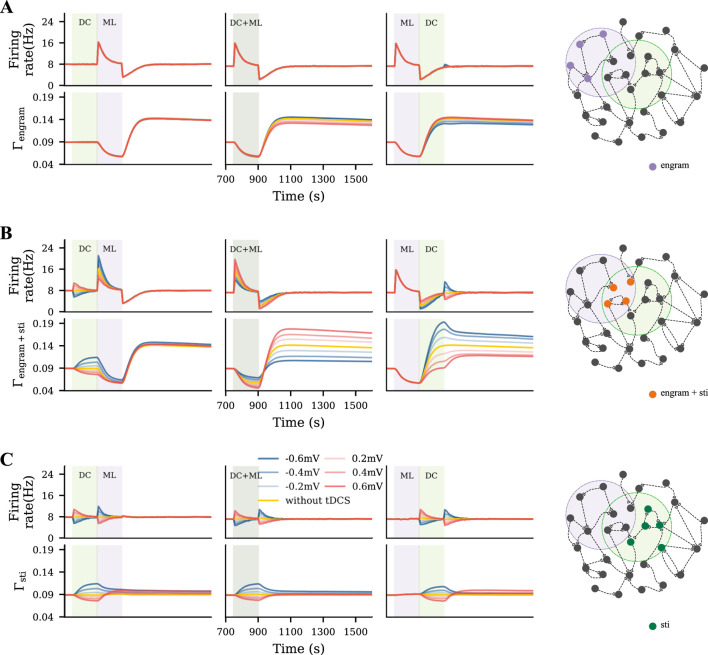
Unfocused tDCS of different intensities applied before, during, or after learning triggered different results. **(A–C)** Temporal evolution of firing rate and connectivity of the population that only sees motor learning input, the overlapped part of the motor engram that was also stimulated, and the neurons only subjected to tDCS, respectively.

To further explore the polarity, intensity, and phase-dependent effects of tDCS in the overlapping scenario, we also performed a systematic analysis. We plotted the temporal evolution of the firing rate and connectivity of the non-overlapped part of the motor engram, the overlapped part of the motor engram, and the tDCS-stimulated cell assembly separately in Figure 6. The non-overlapped half of the engram was slightly affected by tDCS (Figure 6A). The non-overlapped part of the tDCS-stimulated cell assembly was not much influenced by the learning process (Figure 6C). In contrast, the overlapped engram was modulated by tDCS in a contrary way compared to the non-overlapped half (Figures 6B,C). In general, our simulation results showed that weak and strong cell assemblies interacted with each other. The connectivity of the strong cell assembly (motor engram in this case) could be reduced by the following overlapping weak input, due to partial activation, but the connectivity of a weak cell assembly (the tDCS-stimulated cell assembly) is not drastically changed by the following strong input.

### Strong unfocused tDCS distorts the motor engram

**FIGURE 7:**
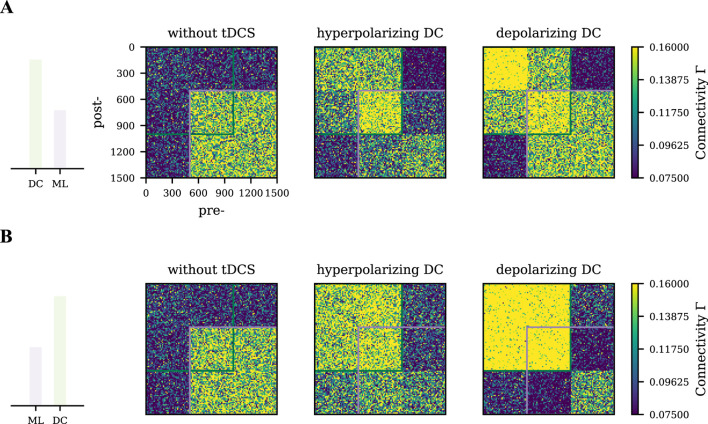
Differential effects of strong tDCS on the motor engram connectivity pattern. Network connectivity was captured at 1200s when the network dynamics reached its equilibrium state. **(A)** Strong tDCS (hyperpolarizing or depolarizing) was applied before motor learning. The green square labels the connectivity of the tDCS-stimulated cell assembly, while the purple square labels the connectivity of the motor engram. Strong hyperpolarizing and depolarizing tDCS resulted in a different connectivity pattern as compared to the control case where only motor learning input was applied but no tDCS (left panel). **(B)** Strong tDCS of both polarities was applied after motor learning. Drastic changes in connectivity pattern were observed in the depolarizing tDCS, where the overlapped engram formed a strong connectivity but almost disconnected with the non-overlapped engram.

As mentioned above, weak unfocused tDCS modulates motor learning in a phase-dependent manner. Then we wondered if a relatively stronger tDCS, compared to motor learning input, would yield similar results. We used tDCS to achieve a ±2.8 mV membrane potential and applied it in an unfocused way before or after the motor learning process. As shown in Figures 7A,B, the motor engram at the time when the network reached its equilibrium state (1200s) showed distinct patterns. Compared to the homogeneous motor engram in which no tDCS was applied, strong hyperpolarizing and depolarizing DC—applied either before or after learning—induced local reorganization within the motor engram. The distorted motor engram may not necessarily represent the same learned input regardless of the averaged connectivity.

## Discussion

Motivated by the empirical observations that tDCS triggered changes in both synaptic transmission and dendritic spine density (Barbati et al., 2020; Jog et al., 2023), we used a neural network model that implements homeostatic structural plasticity in the present study to systematically explore the interference between tDCS and motor learning. Learning input to the network triggers specific remodeling and induces a memory engram that reflects an altered or entirely new motor program as modeled before (Gallinaro et al., 2022). Transcranial DC stimulation also alters the dynamics of the network and induces structural plasticity. However, when applied with different electrode montages and at different phases of motor learning, tDCS interferes with motor learning and modulates the connectivity of the motor engram in specific ways.

**FIGURE 8:**
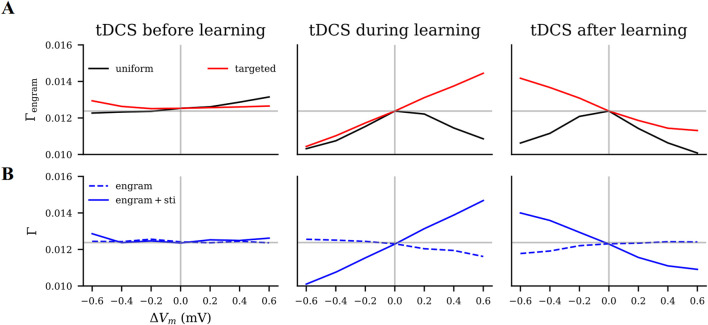
Modulatory effects of tDCS on motor learning as predicted by our model. We estimated the effects of tDCS by the connectivity of the motor engram $\left(\Gamma_{\text{engram}}\right)$ . The $x-\text{axis}(\Delta V_{\text{m}})$ denotes the polarity and intensity of the applied DC. The horizontal solid lines (light gray) indicate the engram’s baseline connectivity without tDCS. **(A)** Effects of uniform and targeted tDCS on engram connectivity when applied immediately before, during, or immediately after learning. **(B)** Connectivity of the overlapped half and the non-overlapped half of the motor engram, when tDCS with different polarities and intensities was applied either before, during, or after learning.

As summarized in Figure 8, both the timing with respect to learning and the focality of stimulation influenced the direction of the effects. Transcranial DC stimulation applied before learning showed generally weak modulatory effects. When administered concurrently with learning, the focality of stimulation played a critical role: Targeted anodal tDCS increased the engram’s connectivity, but targeted cathodal or uniform tDCS regardless of the polarity reduced it in an intensity-dependent manner. Given that the engram’s final connectivity is positively correlated with its evoked response (Gallinaro et al., 2022), such wide-sense “associative” effects of tDCS on motor learning have been observed before. We also predicted with our simulations that, when applied immediately after learning, uniform tDCS generally reduced the connectivity of the engram, but targeted tDCS showed reversed polarity-dependent modulatory effects: anodal tDCS weakened the memory engram, but cathodal stimulation strengthened it. Furthermore, unfocused tDCS affected the connectivity within the memory engram non-uniformly. In summary, our homeostatic plasticity model provides a systematic framework to account for all the confounding factors and mixed results regarding the interaction between tDCS and learning.

### Importance of focality and electrode montage

Large electrodes were confirmed to induce less focal electric field distribution than small button-like electrodes used in the high-definition montage (Cole et al., 2018). However, the definition of focality that we discussed in the current study is a relevant concept with regard to the neural population engaged by a certain task. We classified it by the degree of overlap that the stimulated neuron population has with the neuron ensemble that encodes a motor engram. Our results confirm that the focality of the stimulus is critical to achieving a substantial modulatory effect of tDCS (Edwards et al., 2013; Kuo et al., 2013; Lu et al., 2019; Iannone et al., 2022). Clearly, the three classes of montage considered in our present work do not cover all variants used in motor learning experiments. Unilateral, bilateral, and high-definition electrode layouts are commonly used (Goodwill et al., 2013; Cole et al., 2018), and have been shown to result in mixed effects on motor tasks and learning phases.

We were able to show that uniform stimulation systematically reduces the effect of learning, if applied during or after learning, but will slightly boost learning if applied before learning as anodal stimulation. Notably, uniform stimulation simulated in the present work suggested a scenario in which the induced electric field covers more neurons or a larger area than the motor engram. This is a plausible situation in tDCS practice where large electrode pads induce diffuse electric fields in the brain (Bikson et al., 2010; Bikson et al., 2012). However, these hypothetical predictions do not always agree with the empirical observations. For example, unilateral or bilateral tDCS has been observed in experiments to increase the precision of motor tasks in a follow-up experiment, regardless of stimulation timing (Goodwill et al., 2013). Stimulation before learning using a bilateral montage facilitated learning more than in unilateral montages (Naros et al., 2016). This disagreement might be explained by how the learned skills are represented by the memory engram. The memory engram or motor engram has been demonstrated to be controlled by a group of neurons (Ramirez et al., 2013; Tonegawa et al., 2015) or even a specific population of synaptic spines (Hayashi-Takagi et al., 2015). Integration of new neurons into the memory engram has been proposed to degrade memory and explain infantile amnesia (Akers et al., 2014), and co-activation of neighboring neurons is considered to create false memories (Ramirez et al., 2013; Tonegawa et al., 2015). When uniform anodal tDCS is applied before motor learning, it “primes” the learning and eventually boosts the motor engram connectivity, which could explain the enhanced learning performance in practice. However, uniform tDCS applied during or after learning, regardless of polarity, interrupts motor engram formation by including more neurons than needed into the engram or by reducing motor engram connectivity. This may explain the reduced learning performance observed in practice.

In contrast, we also showed that targeted stimulation either boosts or reduces the impact of learning in a predictable way, depending on the current polarity and phase of the stimulus. Targeted stimulation could, for instance, be achieved by using focused stimulation such as HD-tDCS of the target region. In fact, HD-tDCS has been shown to have better effects in facilitating motor learning (Cole et al., 2018). Animal studies also confirmed that if tDCS precisely modulated the activity of the task-specific neural population, concurrent anodal tDCS restored motor learning in a task-specific manner (Wang et al., 2023). Alternatively, hypothetical engram-specific targeted stimulation would have the strongest effect. The distributed nature of engrams, however, renders this option unrealistic. Some studies have tried to modulate activity in specific neuronal populations when combined with neuroimaging techniques like functional MRI (fMRI), or functional near-infrared spectroscopy (fNIRS), or electroencephalography (EEG) in open-loop or closed-loop configurations (Gao et al., 2021; Martens et al., 2021), or in combination with numerical optimization approaches (Wagner et al., 2016).

### Relative intensity of tDCS and motor learning

In human applications, tDCS is widely accepted as a weak and sub-threshold stimulation method (Jamil et al., 2017). This is the reason we assumed that tDCS provides a weaker stimulus than motor learning in our study. Our model suggests that the relative strength of tDCS compared to motor learning input is critical.

We started with weak transcranial DC stimulation (maximally at 0.6mV ) compared to motor learning (equivalent to 1.5mV ). As suggested for the example of unfocused populations (Figure 5), a strong motor engram is weakened by a weak depolarizing stimulation following it, but not strongly influenced by a weak stimulus applied before it. However, targeted or uniform tDCS of either polarity applied before learning presented weak but facilitating effects (Figure 8A, left panel), which seems to contradict the diminishing effects of tDCS applied before motor learning observed in human experiments (Kuo et al., 2008; Tecchio et al., 2010; Stagg et al., 2011; Apolin et al., 2016; Parma et al., 2021; Rivera-Urbina et al., 2022). Consequently, we examined what happens if the transcranial stimulus is stronger than the motor learning input (±2.8mV) in an unfocused montage (Figure 7). Indeed, strong tDCS applied before or after learning distorted the motor engram’s connectivity pattern. Although strong tDCS enhanced the overlapped engram’s connectivity, the ultimate learning performance that may count on the whole motor engram does not necessarily improve. Strong tDCS which fails to exclusively cover the motor engram will eventually distort the memory information.

### Relative timing of tDCS and motor learning

Another factor confirmed in our model to mediate the modulatory effects of tDCS is the relative timing of tDCS applied to motor learning. Such results have been frequently observed in human experiments and have been summarized as state-dependent stimulation effects (Bradley et al., 2022; Qi et al., 2022; Lapenta et al., 2024). For example, in the case of pre-learning tDCS, anodal stimulation reduced the learning rate, while cathodal stimulation showed facilitation or impairment (Antal et al., 2008; Tecchio et al., 2010; Stagg et al., 2011; Apolin et al., 2016; Parma et al., 2021; Rivera-Urbina et al., 2022). Further studies showed that the impairment caused by pre-learning anodal tDCS was accompanied by a homeostatic increase of ${\text{GABA}}_{\text{A}}$ activity (Amadi et al., 2015). On the basis of these observations, homeostatic plasticity gained popularity as a potential explanation. The idea is that excitability-enhancing tDCS triggers homeostatic inhibition and impairs task performance (Antal et al., 2008). On the other hand, concurrent tDCS showed associative effects: Anodal tDCS usually results in improved learning, while cathodal tDCS does not achieve similar results (Stagg et al., 2011; Ammann et al., 2016; Buch et al., 2017). Concurrent anodal tDCS improves the learning effects not only when applied during the performance of a motor task, but also during observational learning of motor tasks (Wade and Hammond, 2015). Such boosting effects of simultaneous anodal tDCS were mostly discussed as non-homeostatic phenomena, as they could not be explained by homeostatic models (Karabanov et al., 2015; Kronberg et al., 2020).

Compared to other proposed homeostatic or Hebbian models, our HSP model can consistently explain both types of phenomena. Anodal pre-learning tDCS may hinder learning, when applied uniformly, or at an intensity that out-weights motor input. This is considered a homeostatic phenomenon. In contrast, it is considered a Hebbian phenomenon that concurrent anodal tDCS could facilitate learning. In addition, our model provides a systematic explanation for contradictory or confusing results in tDCS studies with different protocols. The electrode montage of tDCS, for instance, influences the relative coverage of the target region by the electric field, and this different focality can induce opposite effects, even if the polarity and intensity of the stimulus remain the same.

### Memory encoding and consolidation

In addition to pre-learning and concurrent stimulation, our work also predicted the effects of post-learning tDCS on memory consolidation. Uniform stimulation applied post-learning or strong unfocused stimulation hindered memory consolidation, but targeted cathodal tDCS applied to the same brain region where motor learning occurs should boost memory consolidation.

Experimental results on the memory encoding and consolidation effects of post-learning tDCS are lacking. Some studies reported that tDCS applied to M1 facilitates memory encoding (Kim et al., 2024) or consolidation (Koyama et al., 2015; Rroji et al., 2015), where the stimulus was administered concurrently with learning, but not after learning. There are also preliminary results showing that the application of anodal tDCS 4h after learning increased the recall of the learned movement sequences in humans (Nitsche et al., 2010). However, the target area was the premotor cortex but not M1. Our predictions based on the HSP model wait to be addressed by experiments. Besides, memory consolidation does not necessarily take place in the same region as motor learning (Rivera-Urbina et al., 2022). Learning and post-learning tDCS might modulate other influencing factors that are relevant to memory consolidation but beyond the scope of our study, such as neural oscillations during sleep (Frase et al., 2020).

### Model selection and limitations

Even though our model has the potential to explain inconsistent tDCS outcomes in the literature, certain choices made in the model require some attention in future work. The first issue concerns the choice of the growth curve. In our present study, we followed the same strategy as in our previous work (Lu et al., 2019) and applied the same linear growth rule for both dendritic spines and axonal boutons. However, the exact shape of the growth curve in a biological setting remains elusive. Computational studies have revealed the distinct properties of different rules, including the linear rule and the Gaussian rules (Butz et al., 2009; Butz and Van Ooyen, 2013; Diaz-Pier et al., 2016; Sinha et al., 2021; Lu et al., 2023). Compared to a linear rule, the Gaussian rules do not favor homeostatic synaptogenesis if the neuron is under the influence of strong chronic inhibition. In contrast, it does not imply any change or even allows for synapse loss, depending on whether the lower setpoint value is at zero or above zero [see Lu et al. (2023)] for comparison of three rules in a similar network setting). Our recent experimental data demonstrated the biological feasibility of a biphasic shape of the growth rule (Lu et al., 2023), which is well approximated by the Gaussian rule in simulations. Although the *non-homeostatic* property of the Gaussian HSP rule is critical to explain the divergent phenomena observed in chronic activity inhibition scenarios (Lu et al., 2023), it does not compromise the use of a linear rule in this study. Given the subthreshold property of tDCS treatment, namely, it excites or inhibits neuronal activity by slightly altering the membrane potential, a linear rule behaves qualitatively similar to a Gaussian rule in this range. Using the linear rule here further enables us to make a direct comparison with our previous tDCS modeling work (Lu et al., 2019).

The same principle applies when deciding whether different rules should be used to govern axonal boutons and dendritic spines. Asymmetric Gaussian-shaped rules—using different setpoint parameters for boutons and spines—in previous work have been considered “more physiological” in response to focal lesion (Butz et al., 2014; Sinha et al., 2021). Indeed, using asymmetric Gaussian rules in combination with distance-dependent connectivity is critical for natural re-wiring after focal lesion (Butz et al., 2014), but not necessarily essential for the after-effects of subthreshold stimulation and inhibition such as tDCS, which requires engram formation during network remodeling. In this regard, the linear rule and Gaussian rules display quite similar homeostatic behavior when modeling the long-term effects of tDCS and allows for engram formation. Therefore, one should not expect any important qualitative differences. We conclude that a model using identical linear rules for pre- and postsynaptic elements is able to capture all essential features.

A third concern is that we assumed the HSP rule and tDCS stimulation selectively to the excitatory, but not inhibitory neurons. This decision was mainly based on empirical evidence obtained from pyramidal cells. The rules of structural plasticity were mainly derived from studies of spine density [see reviews by Holtmaat and Svoboda (2009); Van Ooyen (2011); Caroni et al. (2012)]. Due to their spatially extended morphology, pyramidal cells were considered to be the main target cells of tDCS as well, as the polarization of their membrane is much more pronounced when placed in an electric field (Radman et al., 2009b; Vöröslakos et al., 2018). Nevertheless, computational studies demonstrate that applying HSP to both excitatory and inhibitory neurons can trigger interesting phenomena in response to external perturbations (Diaz-Pier et al., 2016). Studies have also shown that neural modulation can alter synaptic plasticity of inhibitory neurons (Lenz et al., 2016) or activate inhibitory circuits that modulate network dynamics (Anil et al., 2023). Such studies definitely suggest further modeling work to involve inhibitory neurons in plastic responses and neural plasticity.

It should be emphasized that we have only included one structural plasticity rule in the present study, while any forms of functional plasticity, such as spike-timing-dependent plasticity (STDP) and synaptic scaling, may alter the behavior of homeostatic structural plasticity itself. For example, a previous study has shown that the STDP rule could amplify the effects of stimulation and trigger further stronger engram formation (Manos et al., 2021). In contrast, homeostatic synaptic scaling could fine-tune recurrent synaptic weights when stimulated and inhibited, leading to a less prominent change in synapse numbers, which is considered as an indication of structural plasticity (Lu et al., 2023). Therefore, it is worth examining the impact of tDCS in a system with multiple activity-dependent plasticity rules, and the present study with a single plasticity rule can serve as an anchor point for bridging scientific observations.

Last but not least, the formation of engrams among excitatory neurons comes as a secondary effect of network remodeling when the excitatory population of the network is subject to the HSP rule. We use its connectivity to hypothetically represent the after-effects of tDCS or rTMS in this and previous studies (Lu et al., 2019; Anil et al., 2023) as inspired by empirical evidence of memory engram. In learning and memory tasks, for example, engram formation is considered to be elevated connectivity or increased spine density among a group of cells through coactivation (Tonegawa et al., 2015; Hwang et al., 2022). Activated pyramidal cells are confirmed to be the main components of an engram, since neural activation is easily confirmed by the expression of immediate early genes such as c-Fos (Tonegawa et al., 2015; Lu et al., 2021), dendritic morphology can be assessed using imaging techniques (Lu et al., 2021; Lu et al., 2023), and network connectivity can be probed by paired patch clamp (Lenz et al., 2016; Qi et al., 2020). In addition to those, it remains elusive whether inhibition of pyramidal cells leads to the formation of engrams, Furthermore, it has been recently proposed that inhibitory neurons can form a replica of the engram complementing their excitatory counterparts (Barron et al., 2017). Besides, although model parameters that result in increased engram connectivity seem to coincide with parameters that achieve lasting after-effects, there is still a lack of direct experimental evidence that tDCS works through engram formation. One of the reasons is that it is difficult to detect memory engrams experimentally. However, in similar studies, it has been shown that increased engram connectivity leads to more synchronized activity (Gallinaro et al., 2022; Lu et al., 2023), altered network dynamics may therefore serve as an experimental indicator of tDCS after-effects.

## Conclusions and outlook

In the current study, we confirmed again that focal tDCS applied in a targeted manner leads to more predictable and reliable modulatory effects. We also showed that the HSP model can explain both the homeostatic and Hebbian plasticity phenomena observed in experiments using tDCS to modulate motor learning. When applied in a targeted manner or with high intensity, pre-learning anodal stimulation could impair the learning process, whereas concurrent anodal stimulation boosted learning. In addition, we predicted that post-learning cathodal tDCS might facilitate learning, which waits to be addressed in future experiments. Compared to previously proposed plasticity models, the HSP model, which is based on homeostatically controlled spine turnover dynamics, provides a more comprehensive explanation and leads to new predictions about the dynamic interaction between tDCS and motor learning.

But can the HSP rule also explain the tDCS effects observed in other forms of learning? Yes and no. The predictions of our model, such as improved learning for targeted concurrent anodal tDCS, match the results found in verbal learning (Nikolin et al., 2015) and in working memory tasks (Mancuso et al., 2016). Compared to motor tasks, however, verbal learning and working memory require the coordination of more brain regions and demand a model to reflect inter-network communication. Our present study is a first attempt to study the effect of transcranial brain stimulation on complex cognitive processes. To bring more insight to this field, more animal studies are still needed to calibrate the cellular mechanism of tDCS and optimize the homeostatic structural plasticity model.

## Data Availability

The raw data supporting the conclusions of this article will be made available by the authors, without undue reservation.
